# Correlation of MRI-Derived Apparent Diffusion Coefficient (ADC) Values With Gleason Scores in Prostate Cancer and Quantitative Prostate MRI Reading

**DOI:** 10.7759/cureus.106336

**Published:** 2026-04-02

**Authors:** Victoria Y Bird, Krystina Masihy, Juan Varela, Joseph Busch

**Affiliations:** 1 Urology, National Medical Association and Research Group, Gainesville, USA; 2 Urology, HCA Florida North Florida Hospital, Gainesville, USA; 3 Medicine, University of Florida College of Medicine, Gainesville, USA; 4 Radiology, Busch Center, Alpharetta, USA

**Keywords:** adc values, apparent diffusion coefficient (adc), investigation on dwi/adc map, mri guided prostate biopsy, mri prostate, multiparametric prostate mri, prostate biopsy, prostate cancer, quantitative methods, trus-mri fusion biopsy

## Abstract

Background: Accurate characterization of prostate cancer (PCa) aggressiveness remains a clinical challenge. While multiparametric MRI (mpMRI) and Prostate Imaging Reporting and Data System (PI-RADS) scoring are widely used, their qualitative nature introduces inter-reader variability. Apparent diffusion coefficient (ADC) values derived from diffusion-weighted imaging (DWI) may provide a more objective biomarker of tumor severity.

Methods: We retrospectively analyzed 181 lesions from 101 patients with biopsy-proven PCa (2019-2025). The lowest or minimum ADC (ADC_min_) value (µm^2^/s) was recorded for each lesion and correlated with Gleason scores: low-risk (3+3), intermediate-favorable (3+4), intermediate-unfavorable (4+3), and high-risk (≥4+4). Statistical analysis was performed using Welch’s analysis of variance (ANOVA) with Games-Howell post hoc testing.

Results: Mean ADC values showed a progressive decline with increasing Gleason category: 3+3 = 456.5 ± 191 µm^2^/s; 3+4 = 382.0 ± 188 µm^2^/s; 4+3 = 296.2 ± 130 µm^2^/s; ≥4+4 = 222.6 ± 134 µm^2^/s. Interquartile ranges (IQRs) similarly decreased across categories. Welch’s ANOVA demonstrated significant overall group differences (F(3, 84.78) = 18.95, p < 0.001). Games-Howell post hoc tests revealed significantly lower ADC values in 4+3 and ≥4+4 compared with 3+3 (p < 0.001) and in ≥4+4 compared with 3+4 (p < 0.001).

Conclusion: MRI-derived ADC values inversely correlate with Gleason scores, suggesting their potential as a non-invasive biomarker for PCa aggressiveness. Incorporating ADC values into clinical workflows may enhance biopsy targeting, risk stratification, and treatment planning.

## Introduction

Prostate cancer (PCa) is the second most common cancer among men worldwide and a leading cause of cancer-related mortality [1]. Accurate assessment of tumor aggressiveness is central to guiding treatment decisions, ranging from active surveillance for indolent disease to radical prostatectomy or radiation for aggressive tumors.

The Gleason grading system remains the gold standard for stratifying PCa severity and continues to guide both prognosis and management [2,3]. Serum prostate-specific antigen (PSA) testing, while widely used, lacks specificity and may lead to overdiagnosis and overtreatment [4]. Multiparametric MRI (mpMRI) has emerged as an essential tool for lesion detection, with the Prostate Imaging Reporting and Data System (PI-RADS) scoring system facilitating standardized reporting [5,6]. Yet, as a qualitative system, PI-RADS is susceptible to inter-reader variability [7].

Quantitative imaging biomarkers such as the apparent diffusion coefficient (ADC), derived from diffusion-weighted imaging (DWI), offer an opportunity to overcome these limitations. Lower ADC values reflect restricted water diffusion and have been associated with higher tumor cellularity and aggressiveness [8]. While imaging biomarkers such as ADC provide valuable quantitative information, clinical risk stratification for PCa typically incorporates multiple parameters, including prostate-specific antigen density (PSAD), PI-RADS scoring, and histopathology. Therefore, ADC values are best considered as complementary to established clinical and imaging tools rather than standalone predictors. 

This study aimed to assess the relationship between MRI-derived minimum ADC (ADC_min_) values and Gleason score categories in a cohort of patients with biopsy-proven PCa.

## Materials and methods

This retrospective observational study was conducted in accordance with the Strengthening the Reporting of Observational Studies in Epidemiology (STROBE) guidelines [9]. Institutional review board approval was obtained from WIRB-Copernicus Group Institutional Review Board (WCG IRB), with a waiver of informed consent due to the retrospective nature of the study.

A total of 101 patients with histopathology-confirmed PCa who underwent mpMRI followed by MRI-ultrasound fusion-guided prostate biopsy between January 2019 and March 2025 at Urologic Integrated Care, Gainesville, USA, were included in this retrospective study. Patients were identified through review of the electronic health record. Only patients with available prostate MRI, subsequent biopsy-confirmed PCa, and corresponding imaging and histopathologic data were included. Patients with incomplete imaging, inadequate image quality, or missing clinical data were excluded. All MRI examinations were performed before biopsy as part of the standard clinical evaluation for suspected PCa. Both biopsy-naïve patients and patients with prior biopsy were eligible. Patients on active surveillance were included only if they had an MRI followed by biopsy-confirmed PCa during the study period. Prostate biopsies were performed using MRI-ultrasound fusion guidance through either transrectal or transperineal approaches, according to clinical anatomy and surgeon preference. MRI findings were correlated with biopsy results by anatomic lesion localization.

MRI examinations were performed at an outpatient imaging facility using two scanners over the study period: a 3.0T GE system (GE HealthCare, Chicago, USA) before November 2022 and a 1.5T Siemens system (Siemens Healthineers AG, Forchheim, Germany) thereafter. Imaging protocols included T2-weighted imaging and DWI, consistent with standard prostate mpMRI protocols. DWI was acquired using b-values of 50, 100, 300, 800, and 1000 s/mm^2^, with an automatically generated b1400 image. Imaging analysis was performed using GE Invivo DynaCAD Prostate Software (version 5.0; GE HealthCare, Chicago, USA) with concurrent review of axial and coronal T2-weighted images, DWI, and ADC maps. Lesions were manually segmented and contoured in MIM Software (version 7.2.3; MIM Software Inc., Cleveland, USA) to generate three-dimensional regions of interest (ROIs). For each lesion, ADC_min_ (µm^2^/s) within the ROI was recorded. Because this was a retrospective imaging-pathology correlation study, lesion matching was based on radiologic-pathologic anatomic concordance rather than direct voxel-wise co-registration.

Unlike the qualitative PI-RADS, which is subject to inter-reader variability, the ADC provides a quantitative and reproducible metric for tissue characterization [10]. ADC values are derived from diffusion-weighted MRI and reflect the degree of water molecule mobility within tissue. Malignant lesions typically demonstrate restricted diffusion, appearing hyperintense on DWI and corresponding to lower ADC values [11].

ADC maps were generated from DWI sequences, with each voxel (approximately 0.5 × 0.5 × 2 mm) representing a discrete measurement of tissue diffusivity. The use of ADC_min_ was chosen to minimize the influence of partial volume effects and inclusion of non-tumoral components (e.g., vascular structures), which may artificially elevate mean ADC values. A total of 181 lesions were identified and analyzed across the study cohort.

Histopathological analysis from prostate biopsy specimens was used as the reference standard. Lesions were stratified into Gleason grade groups as follows: low-risk (Gleason score 3+3), intermediate-favorable (3+4), intermediate-unfavorable (4+3), and high-risk (≥4+4) [3,12,13].

Continuous variables were summarized using mean ± standard deviation (SD) and interquartile range (IQR), as appropriate. Group differences in ADC values across Gleason categories were assessed using Welch’s analysis of variance (ANOVA) due to heterogeneity of variances, which was confirmed using Levene’s test. Post hoc pairwise comparisons were conducted using the Games-Howell test. A two-tailed p-value < 0.001 was considered statistically significant. All statistical analyses were performed using JASP Software (version 0.19.3; JASP Team, Amsterdam, the Netherlands).

## Results

ADC values derived from DWI demonstrated a stepwise decrease with increasing Gleason grade, indicating an inverse relationship between ADC values and tumor aggressiveness. These values corresponded to measurements obtained using the 1.5T Siemens MRI system and represented scanner-specific ADC characteristics for this cohort. Detailed ADC values by Gleason category are presented in Table 1. The distribution of lesions across Gleason grade groups was as follows: Gleason 3+3 (N = 74, 40.9%), Gleason 3+4 (N = 46, 25.4%), Gleason 4+3 (N = 29, 16.0%), and Gleason ≥4+4 (N = 32, 17.7%).

**Table 1:** Summary of ADC values by Gleason score category ADC values decreased stepwise with increasing Gleason category. The data is represented as N, % from total, mean, SD, IQR Min, and IQR Max. ADC: apparent diffusion coefficient; SD: standard deviation; IQR: interquartile range; Min: minimum; Max: maximum

Gleason group	N	%	Mean ADC (µm^2^/s)	SD	IQR Min	IQR Max
3+3	74	40.9%	456.5	190.6	310	610
3+4	46	25.4%	382	187.8	245	500
4+3	29	16.0%	296.2	129.7	210	385
≥4+4	32	17.7%	222.6	134.2	130	300

**Figure 1:**
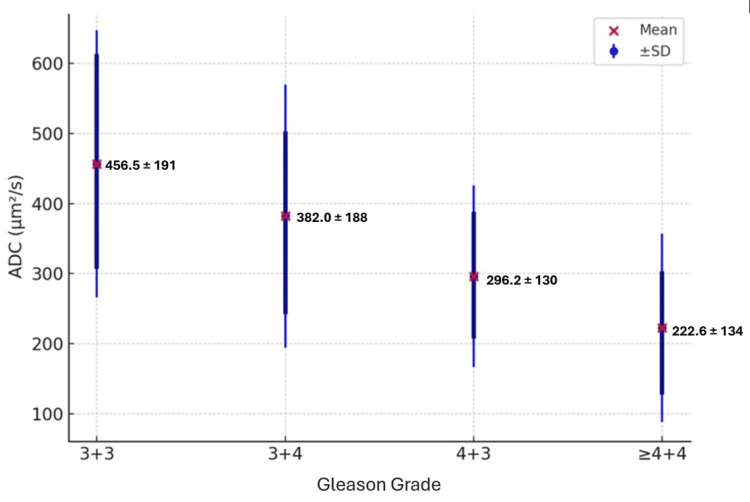
Distribution of minimum ADC values across Gleason categories Distribution of minimum ADC values across Gleason grade groups. The x-axis represents Gleason categories (3+3, 3+4, 4+3, and ≥4+4), while the y-axis denotes ADC values (µm^2^/s). Red markers indicate the mean ADC values for each group, labeled as follows: 456.5 ± 191 µm^2^/s for Gleason 3+3, 382.0 ± 188 µm^2^/s for Gleason 3+4, 296.2 ± 130 µm^2^/s for Gleason 4+3, and 222.6 ± 134 µm^2^/s for Gleason ≥4+4. Blue error bars represent ±1 SD from the mean, and vertical black bars indicate the IQR for each group. The figure demonstrates a progressive decrease in ADC values with increasing Gleason grade, consistent with an inverse relationship between ADC and tumor aggressiveness. ADC: apparent diffusion coefficient; SD: standard deviation; IQR: interquartile range

Group differences in ADC values were assessed using Welch’s ANOVA, which demonstrated a statistically significant difference across Gleason categories (F(3, 84.78) = 18.95, p < 0.001). Post hoc pairwise comparisons using the Games-Howell test revealed significantly lower ADC values in Gleason 4+3 and Gleason ≥4+4 lesions compared with lower-grade groups. These results are summarized in Table 2. Overall, ADC values were inversely correlated with Gleason score, with progressively lower ADC values observed in higher-grade tumors (Figure 1). These findings reflect unadjusted group-level associations between ADC_min_ values and Gleason grade.

**Table 2:** Pairwise comparisons of ADC values (Games-Howell test) Gleason grade group comparison based on mean values of ADC. P-value was considered significant at < 0.001. ADC: apparent diffusion coefficient

Comparison	P-value	Significance
3+3 vs 3+4	> 0.05	Not significant
3+3 vs 4+3	< 0.001	Significant
3+3 vs ≥4+4	< 0.001	Significant
3+4 vs 4+3	> 0.05	Not significant
3+4 vs ≥4+4	< 0.001	Significant
4+3 vs ≥4+4	> 0.05	Not significant

**Figure 2:**
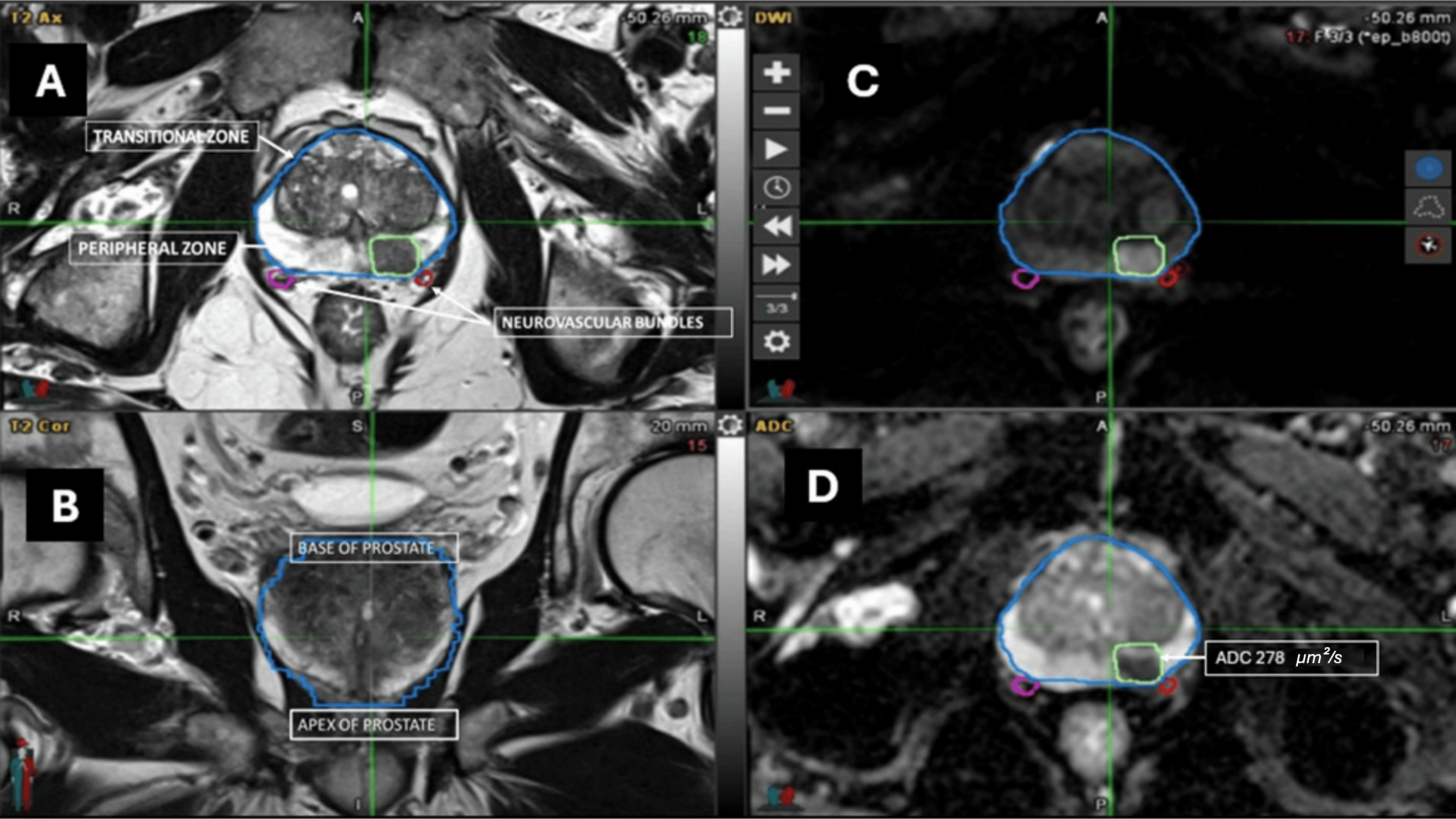
Representative prostate MRI of a lesion with Gleason 4+4 prostate cancer In this patient example, the panels show an axial T2-weighted image (A), a coronal T2-weighted image (B), a diffusion-weighted image (C), and an ADC map (D). In this image series, the prostate is outlined in blue and the lesion is encircled in green. The lesion’s minimum ADC was 278 µm^2^/s. Histopathology demonstrated Gleason 4+4=8, pT3a disease, with positive extracapsular extension (+ECE) and a Decipher grading of high risk (0.75-1.0). ADC: apparent diffusion coefficient

Figure 2 illustrates a representative lesion located in the left paramedian peripheral zone of the prostate, classified as PI-RADS 5 (version 2.1) [6]. The lesion demonstrated an ADC_min_ value of 278 µm^2^/s and corresponded to Gleason 4+4 on histopathological analysis.

## Discussion

This study demonstrates a clear, stepwise decrease in ADC_min_ values with increasing Gleason grade in patients with PCa, highlighting an inverse relationship between ADC values and tumor aggressiveness [14-16]. Higher-grade lesions consistently exhibited lower ADC_min_ values, reflecting increased cellular density and restricted water diffusion, consistent with prior studies [8]. For example, Hambrock et al. reported a median ADC decrease of 0.18 × 10^-3^ mm^2^/s with each increase in qualitative grade group, while De Cobelli et al. observed progressively lower mean ADC values for Gleason scores 6, 7, and 8-10 (p < 0.001) [15,16]. These findings confirm that ADC_min_ provides a robust quantitative biomarker correlating with histopathologic severity [8].

Our results support the utility of ADC values for assessing ROIs and for correlating PI-RADS lesion severity with histopathologic outcomes [17,18]. ADC analysis may aid lesion characterization, targeting during biopsy, and provide additional information for clinical decision-making, including selection between active surveillance and definitive therapy [19-21]. The negative correlation between ADC values and PI-RADS categories observed in previous studies (e.g., Gaur et al., τ = -0.34, p < 0.0001) reinforces the diagnostic relevance of ADC as an adjunct to qualitative PI-RADS evaluation [22].

A distinctive aspect of this study is the focus on the ADC_min_ value per ROI, rather than the mean ADC. This approach captures the most restricted diffusion within a lesion, which better reflects the biologically aggressive tumor components [22,23]. Prior studies have demonstrated that lower percentile ADC values, including the 10th percentile or ADC_min_, outperform mean ADC for predicting clinically significant PCa [23-25]. Donati et al. reported that the 10th percentile ADC yielded higher diagnostic performance (area under the curve (AUC) = 0.758) compared to mean ADC (AUC = 0.704) in differentiating Gleason 6 from ≥7 tumors (p = 0.0001) [23]. Similarly, Wang et al. found that ADC_min_ was the most accurate predictor for clinically significant disease in PI-RADS 3 lesions, serving as an independent predictor of higher-grade pathology [25]. These findings align with our methodology, which prioritizes ADC_min_ as a more precise marker of tumor aggressiveness and prognostic potential [24]. Extremely low ADC values also serve as reliable indicators of PI-RADS severity and overall disease burden [11,26]. Studies by Ataş et al. and Meyer et al. demonstrated that lower ADC values predict higher-risk disease and worse outcomes, including shorter overall survival and higher likelihood of progression [11,26]. Our cohort corroborates these observations, suggesting that ADC_min_ can enhance the stratification of clinically significant lesions and may provide complementary information to conventional imaging assessment [24].

This approach addresses a common limitation of using mean ADC values, which can be skewed by heterogeneous tissue composition within the ROI [23,24,27]. Averaging across voxels may dilute the contribution of highly aggressive tumor foci, potentially underestimating disease severity [24]. By emphasizing ADC_min_, we capture the regions with the most restricted diffusion, providing an accurate reflection of tumor aggressiveness and supporting correlation with histopathologic grade [23,24,27]. Even so, utilizing ADC_min _values carries its own limitations: they may be more susceptible to imaging noise, susceptibility artifacts, and scanner-related variability compared to mean ADC metrics, which may influence measurement precision.

This approach of emphasizing extreme values rather than averages is well-supported across imaging modalities. Just as ADC_min_ highlights the regions of greatest diffusion restriction within a lesion, prostate-specific membrane antigen PET maximum standardized uptake value (PSMA PET SUV_max_) identifies the most metabolically active tumor components, which drive disease aggressiveness [11,28]. Combining these metrics has been shown to improve risk stratification, with ratios such as SUV_max_/ADC providing higher predictive accuracy than either measure alone [26]. Focusing on the extremes captures the biologically most aggressive areas, avoids dilution by surrounding less aggressive tissue, and may better reflect the tumor’s true potential for progression and adverse outcomes [11,28]. This principle reinforces the value of ADC_min_ as a potential quantitative imaging biomarker that may complement existing clinical and imaging tools in guiding biopsy targeting, informing treatment decisions, and ultimately supporting patient management through further characterization of areas of concern [11,28].

Beyond baseline ADC values, changes in ADC over time (ΔADC) have been used to evaluate tissue changes after treatment, providing additional information on tumor response [29,30]. This complements our findings with ADC_min_, highlighting how quantitative diffusion metrics can capture both tumor aggressiveness and dynamic tissue changes [29,30].

Clinically, ADC_min_ analysis is highly accessible and can be implemented using standard imaging software without the need for specialized tools. While ADC analysis does not replace PI-RADS, it may complement lesion characterization, support biopsy targeting, and contribute to treatment planning decisions [17,18]. Integration with advanced imaging analytics, including radiomics and machine learning, may further improve predictive accuracy and facilitate the development of standardized ADC thresholds for clinical use [24].

This study has several limitations. The retrospective, single-institution design and modest sample size may limit generalizability. Variability in MRI scanners (3T vs 1.5T) may influence ADC values, as each system has a unique “ADC signature” [24]. Retrospective normalization of ADC values can partially mitigate this, but scanner-specific differences remain a potential confounder [24]. While the observed inverse relationship between ADC_min_ and Gleason grade is robust, this study reflects unadjusted, retrospective associations. Established clinical variables such as PI-RADS score and PSAD were not incorporated into a multivariable predictive model. Therefore, the independent predictive value of ADC_min_ cannot be determined from this analysis alone, and prospective studies integrating imaging and clinical parameters are warranted. Future multicenter prospective studies are warranted to validate ADC_min_ thresholds across diverse imaging platforms and patient populations [14,24].

This study demonstrates that ADC_min_ values within ROIs decrease progressively with increasing Gleason grade, providing a potential quantitative biomarker of PCa aggressiveness [15,16]. ADC_min_ may offer superior prognostic value compared to mean ADC and can further support the correlation between PI-RADS-defined regions and histopathologic severity [23]. These findings support the potential clinical utility of ADC_min_ in complementing MRI lesion characterization and providing additional information for biopsy targeting and management decisions in PCa [17,18].

## Conclusions

This study demonstrates that ADC_min_ values derived from prostate MRI decrease consistently with increasing Gleason grade, providing a quantitative reflection of tumor aggressiveness. ADC_min_ represents a reproducible and accessible imaging biomarker that may complement existing clinical and imaging tools. Further prospective studies incorporating multivariable clinical parameters are needed to establish its independent role in risk stratification and clinical decision-making. The results underscore the practical applications of ADC_min_ in clinical practice. Incorporating ADC measurements may enhance lesion characterization, help guide biopsy targeting, and provide additional information for decision-making between active surveillance and definitive therapy. By identifying the areas of highest tumor aggressiveness, ADC_min_ may offer additional precision to complement qualitative imaging assessments, potentially improving the ability to stratify patient risk and personalize management strategies.

Overall, ADC_min_ provides a robust, accessible, and clinically meaningful biomarker for PCa evaluation. Its integration into routine imaging workflows may improve diagnostic accuracy and support more informed treatment planning. These findings reinforce the importance of quantitative imaging metrics in advancing precision medicine for PCa.
